# Enhanced surveillance of hospitalised COVID-19 patients in Europe: I-MOVE-COVID-19 surveillance network, February 2020 to December 2021

**DOI:** 10.2807/1560-7917.ES.2023.28.26.2200669

**Published:** 2023-06-29

**Authors:** Heather Mutch, Johanna J Young, Fatima Sadiq, Angela MC Rose, Josie MM Evans, on behalf of the European COVID-19 hospital surveillance analysis writing group

**Affiliations:** 1 Public Health Scotland, Glasgow, Scotland, United Kingdom; 2 Epiconcept, Paris, France; 3 Faculty of Health Sciences and Sport, University of Stirling, Stirling, United Kingdom; 4 Members of the European COVID-19 hospital surveillance analysis writing group are listed under Acknowledgements

**Keywords:** COVID-19, SARS-CoV-2, surveillance, hospitals, Europe

## Abstract

**Background:**

In early 2020, the I-MOVE-COVID-19 hospital surveillance system was adapted from an existing influenza surveillance system to include hospitalised COVID-19 cases.

**Aim:**

To describe trends in the demographic and clinical characteristics of hospitalised COVID-19 cases across Europe during the first 2 years of the pandemic, and to identify associations between sex, age and chronic conditions with admission to intensive care or high dependency units (ICU/HDU) and in-hospital mortality.

**Methods:**

We pooled pseudonymised data from all hospitalised COVID-19 cases in 11 surveillance sites in nine European countries, collected between 1 February 2020 and 31 December 2021. Associations between sex, age and chronic conditions, with ICU/HDU admission and in-hospital mortality were examined using Pearson’s chi-squared test, and crude odds ratio (OR) estimates with respective 95% confidence intervals (CI).

**Results:**

Of 25,971 hospitalised COVID-19 cases, 55% were male, 35% were 75 years or older and 90% had a chronic underlying condition. Patients with two or more chronic underlying conditions were significantly more likely to die in-hospital from COVID-19 (OR: 10.84; 95% CI: 8.30–14.16) than those without a chronic condition.

**Conclusion:**

The surveillance demonstrated that males, those 75 years or older and those with chronic conditions were at greater risk of in-hospital death. Over the surveillance period, outcomes tended to improve, likely because of vaccinations. This surveillance has laid the groundwork for further research studies investigating the risk factors of hospitalised COVID-19 cases and vaccine effectiveness.

**Key public health message**

**What did you want to address in this study?**

At the beginning of the COVID-19 pandemic, in hospital surveillance of respiratory infection such as influenza, was expanded to include patients with COVID-19, and to monitor the seriousness of their disease. We wanted to understand the characteristics and risk factors for severe disease in patients hospitalised with COVID-19 in nine countries in Europe during the first 2 years of the pandemic.

**What have we learnt from this study?**

We showed that among hospitalised patients with COVID-19, males, patients with two or more other long-term conditions and patients aged 75 years and older, were more likely to have more serious disease. Over the course of the pandemic, the number of hospital admissions and the characteristics of patients were highly variable. This was probably because public health measures, vaccination coverage, circulation of SARS-CoV-2 variants and pressures in hospitals varied widely over time and between countries.

**What are the implications of your findings for public health?**

This surveillance system enabled the collection of data to understand what types of patients were being admitted to hospital with COVID-19 during the pandemic. The system was rapidly adapted by a network of European countries and could be used for similar situations in the future.

## Introduction

The emergence of the novel severe acute respiratory syndrome coronavirus 2 (SARS-CoV-2), causing COVID-19, occurred in late 2019 and was classified as a pandemic on 11 March 2020 [1]. By May 2023, over 275 million SARS-CoV-2 infections had been laboratory-confirmed, and over 2 million deaths had been attributed to COVID-19 in Europe [2]. The rapid implementation of high-quality hospital-based surveillance was necessary to identify individuals at greatest risk of severe COVID-19, and to inform the implementation and evaluation of public health interventions [3].

In April 2020, the European Centre for Disease Prevention and Control (ECDC) published their COVID-19 surveillance strategy [4]. By autumn 2020, the Influenza–Monitoring Vaccine Effectiveness in Europe (I-MOVE) network [5] had rapidly adapted their existing infrastructure to establish a surveillance system for hospitalised COVID-19 cases at 11 different sites across nine European countries: Albania, Belgium, England, France (two sites), Lithuania, Portugal, Romania, Scotland and Spain (two sites). It was envisaged that combining hospital surveillance data using a large-scale multicentre approach, alongside estimating the burden of COVID-19 in European hospitals, would lead to a better understanding of risk factors, and would prepare sites for the estimation of vaccine effectiveness once a vaccine became available [6].

Here, we present the demographic and clinical characteristics of COVID-19 cases admitted to hospitals across nine European countries between 1 February 2020 and 31 December 2021, with the aim of providing a comprehensive description of these patients and to explore the natural history of severe disease. During this period, two notable SARS-CoV-2 variants emerged: the Alpha variant (Phylogenetic Assignment of Named Global Outbreak (Pango) lineage designation B.1.1.7) in winter 2020 [7,8] and the Delta variant (Pango lineage designation B.1.617.2) in spring 2021 [9]. These were subsequently found to have different transmission rates and severity levels [7-9]. We also aimed to identify various demographic and clinical factors associated with admission to intensive care or high dependency units (ICU/HDU) and mortality over this period.

## Methods

### Surveillance overview

We pooled pseudonymised data from 11 surveillance sites in nine European countries collected between 1 February 2020 and 31 December 2021. France and Spain each had two distinct administrative sites. Nine of 11 sites used a sentinel questionnaire-based approach, while England and Scotland did not. In England, 53 hospitals were recruited as sentinel sites that collected data on all hospitalised COVID-19 cases, with linkage to national death registrations to verify in-hospital deaths from any cause. Scotland used a register-based approach, using data linkage to identify hospitalised COVID-19 cases, through a unique identifier. The remaining sites collected sentinel data from a total of 25 different hospitals, with between one and six hospitals per site (Table 1).

**Table 1:** Hospitalised COVID-19 cases from participating sites, I-MOVE-COVID-19 hospital surveillance network Europe, February 2020–December 2021 (n = 25,971)

Country	Region	Participating hospitals	Hospitalised COVID-19 cases		Admission of first reported case		Admission of last reported case	
		n	n	%	Date	Week	Date	Week
Albania	Nationwide	2	2,341	9.0	20 Feb 2020	8	28 Dec 2021	52
Belgium	Nationwide	1	1,683	6.5	21 Feb 2020	8	31 Dec 2021	52
England	Nationwide	53^a^	6,124	23.6	15 Mar 2020	11	4 Dec 2021	48
France	FR-R	5	1,797	6.9	1 Feb 2020	5	30 Nov 2021	48
	FR-V	2	18	0.1	10 May 2020	19	22 Oct 2020	43
Lithuania	Nationwide	2	743	2.9	7 Mar 2020	10	28 Dec 2021	52
Portugal	Nationwide	3	1,437	5.5	13 Feb 2020	7	31 Dec 2021	52
Romania	Nationwide	2	596	2.3	10 Mar 2020	11	28 Dec 2021	52
Scotland	Nationwide	119^a^	3,917	15.1	3 Mar 2020	10	31 Dec 2021	52
Spain	ES	2	1,236	4.8	16 Mar 2020	12	16 Nov 2021	46
	NA	6	6,079	23.4	6 Feb 2020	6	6 Dec 2021	49
Total		197	25,971	100.0	1 Feb 2020	5	31 Dec 2021	52

ES: Spanish surveillance site 1; FR-R: French surveillance site 1; FR-V: French surveillance site 2; I-MOVE: Influenza-Monitoring Vaccine Effectiveness in Europe; NA: Spanish surveillance site 2.

^a^ Patients were randomly selected from these hospitals.

Information on sex, age, date of admission, 17 chronic conditions (asthma, anaemia, asplenia, cancer, dementia, diabetes, heart disease, hypertension, immunodeficiency, liver disease, lung disease, neuromuscular disorder, obesity, renal disease, rheumatic disease, stroke, tuberculosis), pregnancy, smoking status, ICU/HDU admission, requirement for mechanical ventilation and discharge date were collected. Definitions of the chronic conditions were outlined in the generic protocol published in June 2020 [6]. Heart disease was the condition with the most complete data, available for 75.5% (19,620/25,971) of hospitalised COVID-19 cases, while data on tuberculosis were only available for 29.6% (7,697/25,971) of hospitalised COVID-19 cases. Within sites, data completion for chronic conditions ranged from 0% to 96%. Data were sent to the central I-MOVE team for cleaning and then to Public Health Scotland for analysis and preparation of regular surveillance bulletins (six bulletins published on the I-MOVE website) [10].

### Case definitions

Here, a hospitalised COVID-19 case denotes an individual admitted to a participating hospital for at least 24 h between 1 February 2020 and 31 December 2021, with a respiratory sample positive for SARS-CoV-2 infection within 14 days of their admission date. This differs from the ECDC case definition as symptoms data had a poor completion rate [11]. 

SARS-CoV-2 infection was confirmed through a PCR test or the inclusion of the U07.1 International Classification of Diseases (ICD)-10 code [12], indicating laboratory confirmation of COVID-19, within a patient’s diagnosis. A COVID-19 death was defined as a hospitalised COVID-19 case who died during their hospital stay, regardless of the recorded cause of death. This may have overestimated the number of COVID-19 deaths at all sites.

### Selection process

The majority of hospitalised COVID-19 cases originated from England and Scotland. To counteract overrepresentation, random but proportionate samples were taken from both sites for analysis. After random selection, 6,124 COVID-19 cases were included from each reporting hospital in England; this number was similar to the sentinel site with the largest number of hospitalised COVID-19 cases, i.e. NA (Spanish surveillance site 2), n = 6,079 hospitalised COVID-19 cases. To retain the proportion of 1.6:1 English to Scottish cases, 3,917 Scottish hospitalised COVID-19 cases were randomly selected for analysis. No selection was carried out on data collected from other sites. The numbers of hospitalised COVID-19 cases submitted from each site are shown in Table 1.

### Statistical analysis

Demographic and clinical data were summarised using absolute and relative frequencies for categorical variables, and medians and interquartile range (IQR) for numerical variables. The proportion of ICU/HDU admissions, mechanical ventilation and in-hospital deaths, length of stay (LOS) in the hospital or ICU/HDU were determined. Different denominators were used for calculating proportions depending on the number of cases for whom relevant data were available. We examined trends in admissions using the month of hospital admission rather than by peaks of hospitalisations, as the duration of different peaks varied by site and not all sites experienced second and third peaks; these were driven by a minority of sites.

Pooling these results from sentinel sites in addition to national sites resulted in unknown denominators of case numbers within the community. This alongside the considerable differences in reporting between sites prevented inter-site analysis.

To determine the associations between sex, age and number of chronic conditions and the ICU/HDU admission or in-hospital death of hospitalised COVID-19 cases, crude odds ratios (OR) with 95% confidence intervals were calculated using the Pearson chi-squared distribution. The association between sex, age and number of chronic conditions was tested against ICU/HDU admission and in-hospital death. Two-sided p values of < 0.05 were chosen to indicate statistical significance. Analysis was carried out using a combination of Microsoft Excel and R software [13,14].

Hospitalised COVID-19 cases with missing data for demographic characteristics (sex, age, supported living, healthcare worker status, smoking status, pregnancy status), chronic underlying conditions, discharge status, ICU/HDU admission status and mechanical ventilation status were excluded from specific relevant analyses.

## Results

**Figure 1:**
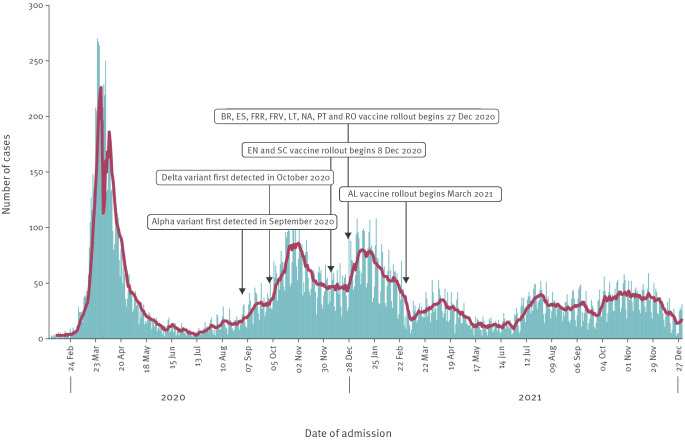
Number of hospitalised COVID-19 cases reported by week of hospital admission, I-MOVE-COVID-19 hospital surveillance network, Europe, February 2020–December 2021 (n = 25,971)

AL: Albania; BE: Belgium; EN: England; ES: Spanish surveillance site 1; FRR: French surveillance site 1; FRV: French surveillance site 2; I-MOVE: Influenza-Monitoring Vaccine Effectiveness in Europe; LT: Lithuania; NA: Spanish surveillance site 2; PT: Portugal; RO: Romania; SC: Scotland.

A total of 25,971 hospitalised COVID-19 cases admitted to the hospital between 1 February 2020 and 31 December 2021 were included in the analysis. In our surveillance, the single day with the largest reported number of hospitalisations was observed in March–April 2020; this sharp peak rose and fell quickly with very low levels of hospitalisations reported in summer 2020. This first peak was observed at most sites, but then slightly different peaks in hospital activity were observed over time at the sites, reflecting the burden across different countries and different variant periods (Figure 1). After this first peak, there were notable increases in the number of reported hospitalised COVID-19 cases during the winter periods of 2020 and 2021.

The red lines denote the 7-day average rolling number of hospitalised COVID-19 cases, whilst the blue lines dictate the case numbers per day.

### Demographic characteristics

Of the 25,971 hospitalised COVID-19 cases, 14,191 (54.6%) were male and 11,780 (45.4%) were female. The median age was 67 years (IQR: 52–79). Patients aged 75 years or older accounted for 34.7% (n = 9,008) of the hospitalisations, while those aged under 45 years accounted for 16.5% (n = 4,292) (Table 2). The median age of hospitalised COVID-19 cases decreased by 5 years over the study period from 69 years (IQR: 59–80) in March 2020 to 64 years (IQR: 47–76) in December 2021.

**Table 2:** Demographic and clinical characteristics of hospitalised COVID-19 cases, I-MOVE-COVID-19 hospital surveillance network, Europe, February 2020–December 2021 (n = 25,971)

Characteristics	SARS-CoV-2-positive hospitalised patients	
	n	%
**Sex**		
Female	11,780	45.4
Male	14,191	54.6
**Age (years)**		
0–44^a^	4,292	16.5
45–64	7,675	29.6
65–74	4,996	19.2
≥ 75	9,008	34.7
Median age (IQR)	67 (52–79)	
**Number of chronic underlying conditions** ^b^		
0	1,763	10.0
1	4,918	27.9
≥ 2	10,971	62.2
Missing^c^	8,319	32.0
**ICU/HDU admission** ^b^		
Yes	3,196	13.7
No	20,093	86.3
Missing^c^	2,682	10.3
Median LOS (IQR) in days	8 (4–18)	
**Mechanical ventilation**		
Any ventilation received^b^	6,754	38.4
- High-flow oxygen^d^	1,608	23.8
- Non-invasive^d^	1,503	22.3
- Invasive^d^	1,007	14.9
- Other^d^	2,323	34.4
- Ventilation type unknown^d^	313	4.6
No ventilation^b^	10,836	61.6
Missing^c^	8,381	32.3
**Discharge status** ^b^		
In-hospital death	4,306	18.8
Discharged from hospital	18,557	81.2
Missing^c^	3,108	12.0
Median LOS (IQR) in days	8 (4–15)	

HDU: high-dependency unit; ICU: intensive care unit; I-MOVE: Influenza-Monitoring Vaccine Effectiveness in Europe; IQR: interquartile range; LOS: length of stay; SARS-CoV-2: severe acute respiratory syndrome coronavirus 2.

^a^ Hospitalised COVID-19 cases aged under 18 years comprised 2.6% (663/25,971) of the surveillance population but these data were not collected by all sites.

^b^ Percentage is calculated as the proportion of hospitalised cases with non-missing data.

^c^ Percentage is calculated as the proportion of all hospitalised cases.

^d^ For types of ventilation used, the percentage is calculated as the proportion of hospitalised cases where any mechanical ventilation was received.

Underlying conditions: asthma, anaemia, asplenia, cancer, dementia, diabetes, heart disease, hypertension, immunodeficiency, liver disease, lung disease, neuromuscular disorder, obesity, renal disease, rheumatic disease, stroke, tuberculosis.

### Chronic conditions and symptoms

Most hospitalised COVID-19 cases (62.2%, 10,971/17,652) reported two or more underlying chronic conditions. There were four chronic conditions that were reported by more than 25.0% of all hospitalised COVID-19 cases: hypertension (43.6%, 7,393/16,968), heart disease (32.6%, 6,396/19,620), obesity (body mass index (BMI) ≥ 30 kg/m^2^; 27.1%, 4,660/17,209), and diabetes (26.1%, 4,996/19,118) (Table 3). The denominators are adjusted for the hospitalised COVID-19 cases with data for each condition. These four conditions were generally among the top five reported chronic conditions across the study sites and over time. Where cases had multiple chronic conditions, the combinations tended to include these four chronic conditions. The most frequently observed combination of co-morbidities was hypertension and heart disease, reported by 33.0% (3,116/9,434) of hospitalised COVID-19 cases with multiple chronic conditions.

**Table 3:** Presence of chronic underlying conditions in hospitalised COVID-19 cases, I-MOVE-COVID-19 hospital surveillance network, Europe, February 2020–December 2021 (n = 25,971)

Conditions	COVID-19 cases (n = 25,971)						ICU/HDU (n = 3,196)						Deaths (n = 4,306)					
	Yes		No		Missing		Yes		No		Missing		Yes		No		Missing	
	n	%^a^	n	%^a^	n	%^b^	n	%^a^	n	%^a^	n	%^b^	n	%^a^	n	%^a^	n	%^b^
Hypertension	7,393	43.6	9,575	56.4	9,003	34.7	1,198	46.9	1,359	53.1	639	20.0	1,765	59.7	1,189	40.3	1,352	31.4
Heart disease	6,396	32.6	13,224	67.4	6,351	24.5	730	26.8	1,995	73.2	471	14.7	1,839	52.3	1,678	47.7	789	18.3
Obesity	4,660	27.1	12,549	72.9	8,762	33.7	939	36.9	1,609	63.1	648	20.3	751	23.9	2,386	76.1	1,169	27.1
Diabetes	4,996	26.1	14,122	73.9	6,853	26.4	800	29.5	1,914	70.5	482	15.1	1,200	35.1	2,221	64.9	885	20.6
Cancer	2,882	16.5	14,536	83.5	8,553	32.9	323	13.3	2,105	86.7	768	24.0	783	24.6	2,401	75.4	1,122	26.1
Asthma	1,828	10.0	16,409	90.0	7,734	29.8	219	8.3	2,414	91.7	563	17.6	364	11.0	2,958	89.0	984	22.9
Lung disease^c^	2,897	15.7	15,538	84.3	7,536	29.0	400	15.0	2,262	85.0	534	16.7	798	24.5	2,465	75.5	1,043	24.2
Renal disease	2,924	15.4	16,051	84.6	6,996	26.9	326	12.0	2,398	88.0	472	14.8	961	28.5	2,416	71.5	929	21.6
Neuromuscular disorder	1,469	10.2	12,901	89.8	11,601	44.7	121	5.4	2,121	94.6	954	29.8	424	16.2	2,195	83.8	1,687	39.2
Stroke	729	6.4	10,729	93.6	14,513	55.9	73	4.6	1,503	95.4	1,620	50.7	216	13.1	1,429	86.9	2,661	61.8
Rheumatic illness	1,068	6.3	15,847	93.7	9,056	34.9	124	5.5	2,132	94.5	940	29.4	282	9.2	2,797	90.8	1,227	28.5
Dementia	912	5.8	14,813	94.2	10,246	39.5	22	1.1	1,936	98.9	1,238	38.7	349	12.2	2,502	87.8	1,455	33.8
Liver disease	972	5.2	17,825	94.8	7,174	27.6	150	5.5	2,557	94.5	489	15.3	211	6.3	3,148	93.7	947	22.0
Anaemia	578	5.0	10,959	95.0	14,434	55.6	86	5.4	1,501	94.6	1,609	50.3	131	7.9	1,520	92.1	2,655	61.7
Immunodeficiency	542	3.2	16,654	96.8	8,775	33.8	91	3.8	2,295	96.2	810	25.3	150	4.8	2,994	95.2	1,162	27.0
Tuberculosis	81	1.1	7,616	98.9	18,274	70.4	15	1.1	1,340	98.9	1,841	57.6	13	1.1	1,137	98.9	3,156	73.3
Asplenia^d^	93	1.0	9,435	99.0	16,443	63.3	< 10	< 1.0	> 1,000	> 99.0	1,945	60.9	27	1.4	1,909	98.6	2,370	55.0

HDU: high dependency unit; ICU: intensive care unit; I-MOVE: Influenza-Monitoring Vaccine Effectiveness in Europe.

^a^ Percentage of hospital admissions with non-missing data.

^b^ Percentage is the proportion with missing chronic condition data.

^c^ Differences between sites meant that asthma was occasionally included in lung disease.

^d^ These values are not presented because of small numbers and to prevent disclosure.

### Intensive care unit/high dependency unit admission

**Figure 2:**
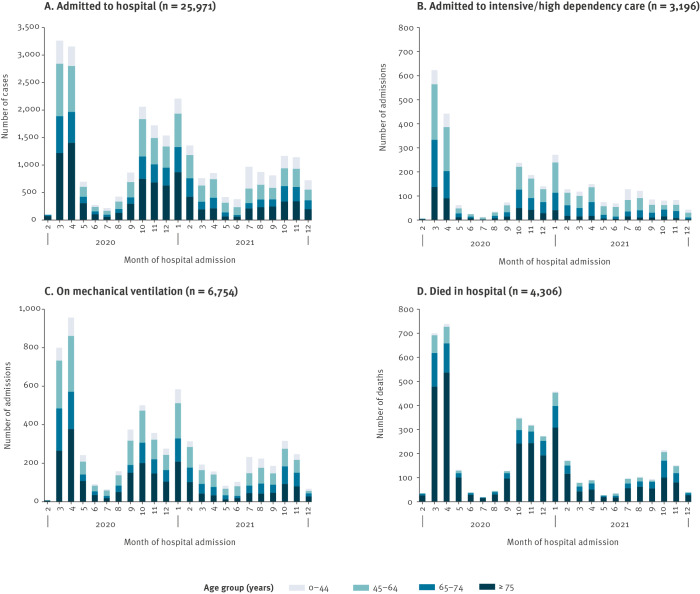
Outcomes of hospitalised COVID-19 cases by age group, I-MOVE-COVID-19 hospital surveillance network, Europe, February 2020–December 2021

Admission to ICU/HDU was explored in 23,289 hospitalised COVID-19 cases where information was available: 16.7% (2,097/12,566) of males and 10.2% (1,099/10,723) of females were admitted to ICU/HDU. Males had higher odds of being admitted to ICU/HDU compared with females. The median age of hospitalised COVID-19 cases admitted to ICU/HDU was 63 years (IQR: 53–72). Hospitalised COVID-19 cases aged 45–64 and 65–74 years had the greatest odds of admission to ICU/HDU, compared with those aged 0–44 years (Table 4), and these patients consistently comprised the largest proportion admitted to ICU/HDU each month (Figure 2B). Hospitalised COVID-19 cases aged 75 years and older had lower odds of being admitted to ICU/HDU when compared with those aged 0–44 years (Table 4).

**Table 4:** Severe outcomes of COVID-19 cases by selected risk factors, I-MOVE-COVID-19 hospital surveillance network, February 2020–December 2021 (n = 25,971)

Exposures (risk factors)	Proportion of hospitalised COVID-19 cases with known ICU/HDU admission							Proportion of hospitalised COVID-19 cases with known outcome						
	Analysis population (n = 23,289)^a^		ICU/HDU (n = 3,196)		Significance			Analysis population (n = 22,863)^b^		Deaths (n = 4,306)		Significance		
	n	%^a^	n	%^b^	OR	OR range	p	n	%^c^	n	%^d^	OR	OR range	p
Sex														
Female	10,723	46.0	1,099	10.2	Reference			10,435	45.6	1,762	16.9	Reference		
Male	12,566	54.0	2,097	16.7	1.75	1.62–1.90	< 0.05	12,428	54.4	2,544	20.5	1.27	1.18–1.36	< 0.05
Age group (years)														
0–44	3,863	16.6	454	11.8	Reference			3,762	16.5	63	1.7	Reference		
45–64	6,909	29.7	1,292	18.7	1.72	1.16–2.58	< 0.05	6,830	29.9	489	7.2	4.53	1.44–14.15	< 0.05
65–74	4,206	18.1	898	21.4	2.04	1.36–3.05	< 0.05	4,171	18.2	857	20.5	15.18	4.86–47.40	< 0.05
≥ 75	8,311	35.7	552	6.7	0.53	0.36–0.80	< 0.05	8,100	35.4	2,897	35.8	32.69	10.49–101.89	< 0.05
Number of chronic underlying conditions														
0	1,762	10.6	234	13.3	Reference			1,744	10.7	57	3.3	Reference		
1	4,284	25.9	652	15.2	1	0.85–1.18	0.23	4,332	26.5	662	15.3	5.4	4.09–7.12	< 0.05
≥ 2	10,515	63.5	1,573	15.0	0.91	0.79–1.06	0.07	10,272	62.8	2,731	26.6	10.84	8.30–14.16	< 0.05

HDU: high dependency unit; ICU: intensive care unit; I-MOVE: Influenza – Monitoring Vaccine Effectiveness in Europe; OR: odds ratio.

^a^ Percentage of COVID-19 cases dependent on the analysis population with known ICU/HDU admission status (n = 23,289), or for those with known ICU/HDU admission status and chronic condition (n = 16,561).

^b^ Percentage of COVID-19 cases admitted to ICU/HDU dependent on the proportion within the analysis population.

^c^ Percentage of COVID-19 cases dependent on the analysis population with known outcome (n = 22,863), or for those with known ICU/HDU admission status and chronic condition (n = 16,348).

^d^ Percentage of COVID-19 cases who died in-hospital dependent on the proportion within the analysis population.

Underlying conditions: asthma, anaemia, asplenia, cancer, dementia, diabetes, heart disease, hypertension, immunodeficiency, liver disease, lung disease, neuromuscular disorder, obesity, renal disease, rheumatic disease, stroke, tuberculosis.

I-MOVE: Influenza – Monitoring Vaccine Effectiveness in Europe.

No association (p > 0.05) was observed between the number of chronic conditions and admission to ICU/HDU (Table 4). However, the prevalence of the most common conditions individually was generally higher in hospitalised COVID-19 cases admitted to ICU/HDU: hypertension 46.9% (1,198/2,557), heart disease 26.8% (730/2,725), obesity 36.9% (939/2,548) and diabetes 29.5% (800/2,714). The denominators are adjusted for the number of hospitalised COVID-19 cases with data for each condition.

Over the entire study period, the overall median LOS within ICU/HDU was 8 days (IQR: 4–18) (Table 2) but decreased from 9 days (IQR: 4–24) in March 2020 to 7 days (IQR: 4–14) in November 2021. Hospitalised COVID-19 cases who required ICU/HDU had a median overall LOS in hospital of 18 days (IQR: 10–32), compared with 7 days (IQR: 4–13) for those who did not require ICU/HDU.

Mechanical ventilation was required by 38.4% (6,754/17,590) of hospitalised COVID-19 cases where information was available, 14.9% (1,007/6,754) required invasive mechanical ventilation. Any method of mechanical ventilation was required by 42.1% (4,076/9,667) of males and 33.8% (2,678/7,923) of females.

### Discharge status

The majority (81.2%, 18,557/22,863) of hospitalised COVID-19 cases with known outcomes were discharged from hospital. The median LOS in hospital for those discharged was 8 days (IQR: 4–15). The median LOS in the hospital tended to be longer in those aged 75 years and older (11 days, IQR: 6–19) and shorter in those aged less than 45 years (5 days, IQR: 2–9). The overall LOS in hospital decreased from 9 days (IQR: 5–16) in March 2020 to 7 days (IQR: 3–12) in November 2021.

### Mortality

Mortality was explored in 22,863 hospitalised COVID-19 cases for whom this information was available. Approximately one-fifth of male hospitalised COVID-19 cases (20.5%, 2,544/12,428) died during their hospital stay compared with 16.9% (1,762/10,435) of females. The median age of hospitalised COVID-19 cases who died in the hospital was 80 years (IQR: 71–87), with 35.8% (2,897/8,100) of those aged 75 years or older dying during their hospital stay (Table 4). Over time, the proportions of hospitalised COVID-19 cases dying in the hospital decreased from 24.8% (737/2,974) in April 2020 to 18.3% (150/820) in November 2021, but proportions were considerably higher in the winter months compared with the summer months (Figure 2D). The median LOS in the hospital for those who died was 10 days (IQR: 5–19).

Of all hospitalised COVID-19 cases admitted to the ICU/HDU, 30.8% (935/3,037) died while in the hospital. Where information on timing of death was available, 34.5% (313/908) died within 7 days of the ICU/HDU admission date and 15.6% (142/908) died more than 28 days from ICU/HDU admission date.

Hospitalised COVID-19 cases with two or more underlying chronic conditions had 11 times higher odds of dying during their hospital stay than those without (Table 4). Hypertension was reported by 59.7% (1,765/2,954) of hospitalised COVID-19 cases who died, followed by heart disease (52.3%, 1,839/3,517) and diabetes (35.1%, 1,200/3,421).

## Discussion

Our surveillance found that a high proportion of hospitalised COVID-19 cases were male, were aged 75 years or older, or had at least one chronic underlying condition, as also reported in numerous studies published globally [15-20]. The most common comorbidities were hypertension, heart disease, obesity and diabetes. Data published within Europe indicate that prevalence estimates of these diseases in adults in the most recent years were: hypertension 22% [21], cardiovascular disease 7% [22], obesity 17% [23] and diabetes 6% [24], considerably lower than the prevalence within our surveillance population. Individuals with chronic conditions are reported to be at higher risk of hospitalisation and severe disease for other respiratory infections such as influenza [25]. The proportion of adults with these diseases varies between different countries and, therefore, unweighted average proportions for Europe must be considered carefully.

A higher proportion of males required admission to the ICU/HDU and mechanical ventilation, which aligns with several international large scale surveillance studies and meta-analyses [16,19,26,27]. Hospitalised COVID-19 cases aged 45–74 years were more commonly admitted to the ICU/HDU than those aged 75 years or older, but the extent of triaging by age is unknown. Age alone is not a reason to prevent older patients from being admitted to ICU/HDU [28]. Times of unprecedented hospital burden, such as the COVID-19 pandemic, put increased pressures on resources, potentially reducing the number of older patients admitted to the ICU in an effort to concentrate scarce resources for the greatest overall patient benefit. This could have reduced the number of patients aged 75 years or older being admitted to ICU/HDU [29].

Prior to the COVID-19 pandemic, the average hospital LOS for any cause of hospitalisation across Europe had decreased from 10 days in 2000 to 7.5 days in 2018 [22,30]. The average LOS in 2018 matches closely with the median LOS in hospital of hospitalised COVID-19 cases over the study period. Our study found that the median LOS was higher for hospitalised COVID-19 cases admitted to the ICU/HDU compared with those who were not. Other studies have also shown that those admitted to the ICU/HDU had longer median LOS than those who were not admitted to the ICU/HDU [31]. This increased LOS is likely the result of these patients experiencing more severe disease.

Throughout the entire study period, approximately one-fifth of hospitalised COVID-19 cases died during their hospital stay. In 2019, the crude proportion of deaths across the European Union was 10%, this increased to 12% in 2020 and 2021, potentially because of the effect of COVID-19 on mortality [32]. The higher proportion of deaths within our study population reflects the greater severity of disease in hospitalised patients but could also have been a result of some selection bias within the hospitals.

A higher proportion of male hospitalised COVID-19 cases died compared with females, as did those with two or more chronic underlying conditions and those aged 75 years or older. The higher proportion of older hospitalised COVID-19 cases dying was reflected in the absolute and relative frequencies but also in the median age of those who died (80 years, IQR: 71–87) compared with those who were admitted to hospital (67 years, IQR: 52–79). Having multiple chronic conditions and increasing age are risk factors for hospital admission and severe outcomes in general [33,34]. The higher proportion of male hospitalised COVID-19 cases dying has been observed elsewhere [16,19,26,27] and has been explained by multiple factors including genetic, immunological, behavioural and social aspects, and that males tend to experience an increased proportion of life threatening/more severe chronic conditions [35] compared with females [36,37].

The highest number of hospitalised COVID-19 cases reported in a single day was observed in March 2020, but this peak decreased rapidly by April and remained low throughout the summer months. This trend was also observed in the proportion of hospitalised COVID-19 cases who died during their hospitalisation. As the pandemic progressed and the incidence of SARS-CoV-2 decreased in the community, partly because of public health interventions such as physical distancing measures, the number of hospitalisations also decreased.

The emergence of the Alpha SARS-CoV-2 variant in winter 2020 was met with higher case numbers compared with the summer months because of increased transmissibility [7,8]. Between January and May 2021, the proportion of hospitalised COVID-19 cases aged 75 years or older decreased from 39% to 15%, which was likely the result of the roll-out of the first COVID-19 vaccines to individuals within this age group.

In spring of 2021, the Delta SARS-CoV-2 variant became dominant in Europe and was reported to show greater transmissibility than the Alpha variant [9]. An increase in the number of reported hospital admissions was observed between June and July 2021. This could have been the result of a combination of relaxed restrictions over summer and the occurrence of major events such as the European Football Championships [38,39]. In addition, the roll-out of vaccines was in progress at this time; younger individuals (aged ≤ 45 years) may not have received their COVID-19 vaccination, while older patients (aged ≥ 75 years) may have experienced vaccine waning [40].

At the end of the study period, the median age of hospitalised COVID-19 cases decreased from 69 years in March 2020 to 64 years in December 2021. This was also reflected in the proportions of hospitalised COVID-19 cases who were living in assisted living accommodation such as care or residential home; this proportion was highest in April 2020 (29.6%) but decreased to 5.1% in April 2021. The proportion of hospitalised COVID-19 cases who died in the hospital varied by month: this was highest in April 2020 at 25.2% and was lowest in May 2021 at 7.1%. This coincided with the vaccine roll-out to older age-groups, which was shown to provide high levels of effectiveness against severe COVID-19 disease (hospitalisation and death) when the complete vaccination course was administered [41,42].

This surveillance has some limitations. Firstly, pooling data from different countries and sites has its own challenges. The numbers of hospitalised COVID-19 cases varied by country, number and size of sentinel sites, as well as by local or national thresholds for hospital admission, and were not necessarily proportionate to the population of the country. Secondly, each site experienced strains of SARS-CoV-2 and applied intervention strategies at different times; this resulted in heterogeneity, which meant that inter-site or peak analysis was too challenging without the exclusion of many records [43]. Thirdly, data collection methods differed between sites, which was a result of approaches taken to circumvent capacity issues brought on by the COVID-19 pandemic. This impacted the data completeness of certain variables resulting in high levels of missing data, e.g. chronic underlying conditions, symptoms, days between symptom onset and hospital admission, vaccination status and virus strain. This may affect the accuracy and generalisability of results [44]. Therefore, this paper did not explore the direct effect of vaccination on the severity of COVID-19. However, this will be explored in a subsequent risk factor and vaccine effectiveness study. Fourthly, numbers quoted for ages were absolute, rather than rates, as the hospital surveillance populations were not known for all sites involved. Fifthly, the proportion of known outcomes for the latter months were lower as a greater number of patient outcomes were unknown at the final data collection. Finally, the number of COVID-19 deaths may be overestimated as cause of death was not used to define these. However, requiring validated cause of death would have compromised the timeliness of the surveillance system.

Despite these challenges, the I-MOVE-COVID-19 surveillance system rapidly mobilised in the face of the pandemic and provided critical knowledge on the natural history of severe COVID-19. Results of this large-scale, cross-collaborative surveillance system suggested that males, patients with two or more chronic underlying conditions and those aged 75 years and older were at higher risk of severe disease. When the Delta variant was predominant in Europe, we observed an increase in the number of reported admissions, suggesting that individuals were more likely to experience severe disease during this variant period. After this surveillance ended, the Omicron variant emerged; this study has shown that it is important to monitor the hospitalised COVID-19 cases to gain an indication of disease severity. However, it is important that this intelligence can be disseminated quickly enough for local and national response. This question is now being addressed in a formal evaluation of the I-MOVE-COVID-19 surveillance system.

## Conclusions

Against the background of a global pandemic, the setup of this European surveillance allowed local and national perspectives to be placed into a wider context. Groundwork was laid for critical risk factor and vaccine effectiveness studies on SARS-CoV-2 infection. This surveillance system informs regional, national, European and global perspectives that will determine the options for long-term surveillance of COVID-19 cases and supports public health professionals who make decisions about thresholds as indicators of increasing cases and the requirement for additional public health interventions.
